# Error in statistical tests of error in statistical tests

**DOI:** 10.1186/1471-2288-6-45

**Published:** 2006-09-13

**Authors:** Monwhea Jeng

**Affiliations:** 1 Department of Physics, Box 1654, Southern Illinois University Edwardsville, Edwardsville, IL 62025; 2 Department of Physics, Syracuse University, Syracuse, NY 13244

## Abstract

**Background:**

A recent paper found that terminal digits of statistical values in Nature deviated significantly from an equiprobable distribution, indicating errors or inconsistencies in rounding. This finding, as well as the discovery that a large percentage of *p *values were inconsistent with reported test statistics, led to a great deal of concern in the popular press and scientific community. The findings ultimately led to new guidelines for all Nature Research Journals.

**Methods:**

We checked the statistical analysis behind the original paper's tests of equiprobability.

**Results:**

The original paper tested equiprobability with the Kolmogorov-Smirnov test outside its regime of validity. Correct tests find no statistically significant deviations from equiprobability for the statistical values in Nature.

**Conclusion:**

Statistical tests should be used correctly.

## Background

A recent paper concluded that "statistical practice is generally poor, even in the most renowned scientific journals" [1]. The paper prompted significant attention in the popular press, and serious concern within the scientific community [2-7]. It led the editors of Nature Medicine to review their statistical practices, ultimately resulting in new statistical guidelines for all Nature Research Journals [3].

One of the two main results of [1] was that terminal digits of statistical values in Nature deviated significantly from an equiprobable distribution, indicating errors or inconsistencies in rounding. The authors of [1] collected random samples of test statistics and *p *values published in Nature, and looked at the terminal digits of these numbers. Their raw data is shown in tables 1 and 2. They argued that these terminal digits should be spread evenly among the ten possible digits. Applying the Kolmogorov-Smirnov test with SPSS for Windows, they obtained *Z *= 2.7, *p *< 0.0005, for the 610 test statistics, and *Z *= 1.4, *p *= 0.043, for the 181 *p *values. They thus concluded that the terminal digits suffered from errors, most likely due to poor rounding procedures. We point out that the original paper's test of equiprobability was based on invalid use of the Kolmogorov-Smirnov test on categorical data and that correct statistical testing finds no statistically significant deviations from equiprobability.

**Table 1:** Terminal digits of test statistics from volumes 409–412 of *Nature*

Digit	Frequency
0	67
1	67
2	65
3	71
4	51
5	58
6	53
7	61
8	62
9	55

**Table 2:** Terminal digits of *p *values from volumes 409–412 of *Nature*

Digit	Frequency
0	10
1	20
2	25
3	24
4	12
5	16
6	25
7	20
8	16
9	13

The authors of [1] also found a number of cases where *p *values in Nature and the British Medical Journal were reported incorrectly, based on comparison with the reported test statistics. That finding is unaffected by our analysis.

## Methods

We ran tests of equiprobability on terminal digits of the test statistics and *p *values in Nature, using both *χ*^2 ^tests, and a modification of the Kolmogorov-Smirnov test for categorical data.

## Results and Discussion

The Kolmogorov-Smirnov test is normally used to test whether data follows a specified *continuous *distribution [8]. A simple calculation from the raw data in [1] shows that the *Z *and *p *values obtained there are those based on comparing the data with a distribution uniform on the continuous interval [0, 9]. But this distribution is obviously incorrect even before any comparison with the data, since the terminal digit cannot be, for example, 2.68. A check of the documentation for SPSS for Windows confirms that the program runs the Komogorov-Smirnov test for a continuous uniform distribution, rather than a discrete uniform distribution.

Because the terminal digits are naturally discrete, a *χ*^2 ^test is appropriate [8]. *χ*^2 ^tests yield *χ*^2 ^= 6.5, df = 9, *p *= 0.69, for the 610 test statistics, and *χ*^2 ^= 15, df = 9, *p *= 0.086, for the 181 *p *values. This changes the results from "significant" to "not significant," and we therefore have insufficient evidence to suggest terminal digit errors in the *p *values reported in Nature articles.

Because the reader may be suspicious that this is simply a judgment call as to the most natural statistical test, rather than a bona fide mistake in [1], we also rerun the Kolmogorov-Smirnov test. While it is unusual to run this test on discrete data, it is possible (although perhaps poorly motivated in this case), so long as appropriate modifications are made. Instead of incorrectly comparing the data against the distribution *P*(*x*) = 1/9 for 0 ≤ *x *≤ 9, we use *P*(*x*) = (1/10)∑j=09δ(x−j)
 MathType@MTEF@5@5@+=feaafiart1ev1aaatCvAUfKttLearuWrP9MDH5MBPbIqV92AaeXatLxBI9gBaebbnrfifHhDYfgasaacH8akY=wiFfYdH8Gipec8Eeeu0xXdbba9frFj0=OqFfea0dXdd9vqai=hGuQ8kuc9pgc9s8qqaq=dirpe0xb9q8qiLsFr0=vr0=vr0dc8meaabaqaciaacaGaaeqabaqabeGadaaakeaadaaeWaqaaGGaciab=r7aKjabcIcaOiabdIha4jabgkHiTiabdQgaQjabcMcaPaWcbaGaemOAaOMaeyypa0JaeGimaadabaGaeGyoaKdaniabggHiLdaaaa@3A20@. This gives *Z *= 1.1 for the 610 test statistics (Δ = 0.043), and *Z *= 0.60 for the 181 *p *values (Δ = 0.045). Since the textbook tables of Kolmogorov-Smirnov *p *values are computed for continuous distributions, we convert from *Z *to *p *values with Monte Carlo simulations (counting ties in *Z *as "half a hit"). This gives *p *= 0.094 and *p *= 0.57 for the two cases. Again, the terminal digit distributions could reasonably have occurred by chance, given a discrete uniform distribution.

The authors of [1] also found that 21 of the 181 *p *values in Nature had problems when compared with the corresponding test statistics. Our analysis does not change this finding, but it is worth remarking on the comparatively minor nature of many of the problems that they found. For example, 3 of the 21 problems come from a single three-row table (table 1 of [9]), in which every entry of a column labelled *P *reads 0.001. This is indeed somewhat misleading, since the natural implication is that *p *= 0.001 for all three entries, when in fact the intended meaning of the table was (presumably) that all three results were significant at the 0.1% level (i.e. *p *< 0.001). But it is hard to imagine that many readers would be badly misled by this table, and in any event, such errors are minor in comparison to the error of using a demonstrably invalid test.

## Conclusion

The authors of [1] concluded that statistical tests in papers need to be inspected more closely. However, one of the main findings of their paper is invalidated by incorrect use of a statistical test. It is ironic that despite the great attention that their paper has attracted over the last two years, this error has escaped notice. While their paper still points to the need for greater scrutiny of statistics, that scrutiny would be better directed at the assumptions used in the statistical tests, rather than at the precise *p *values obtained.

## Competing interests

The author(s) declare that they have no competing interests.

## Authors' contributions

MJ is responsible for this work in its entirety.

## Pre-publication history

The pre-publication history for this paper can be accessed here:


